# Supplementary material for: Detailed molecular and epigenetic characterization of the pig IPEC-J2 and chicken SL-29 cell lines
Source: iScience. 2023 Feb 20;26(3):106252. doi: 10.1016/j.isci.2023.106252 (PMC10018572; doi:10.1016/j.isci.2023.106252)
Supplement: Data S2. Complete homer output for identified motifs in Chicken SL-29, related to Tables 5 and 6 — Homer motif analysis results for histone modifications H3K4me1, H3K4me3, H3K27ac, enhancers, and ATAC-seq of chicken SL-29 cell line. Parameters for possible false positives is as mentioned earlier for S5. [file mmc3.zip › Data_S2/S6/Chicken_SL_29/peak_files_H3K4me3/homerResults/motif2.similar.html]

motif2

## Information for motif2

A
G
C
T
A
G
T
C
A
C
T
G
C
A
G
T
C
G
A
T
C
G
T
A
C
A
G
T
A
G
T
C
  
Reverse Opposite:  

T
C
A
G
G
T
C
A
G
C
A
T
G
C
T
A
G
T
C
A
A
G
T
C
A
C
T
G
T
C
G
A
  

|  |  |
| --- | --- |
| p-value: | 1e-137 |
| log p-value: | -3.172e+02 |
| Information Content per bp: | 1.609 |
| Number of Target Sequences with motif | 10003.0 |
| Percentage of Target Sequences with motif | 58.27% |
| Number of Background Sequences with motif | 15891.5 |
| Percentage of Background Sequences with motif | 48.73% |
| Average Position of motif in Targets | 150.0 +/- 85.1bp |
| Average Position of motif in Background | 149.4 +/- 101.5bp |
| Strand Bias (log2 ratio + to - strand density) | 0.0 |
| Multiplicity (# of sites on avg that occur together) | 1.59 |
| Motif File: | file (matrix) reverse opposite |

### Similar de novo motifs found

|  |  |  |  |  |  |  |  |
| --- | --- | --- | --- | --- | --- | --- | --- |
| Rank | Match Score | Redundant Motif | P-value | log P-value | % of Targets | % of Background | Motif file |
| 1 | 0.846 | C T A G A G C T G T C A A G T C A T C G G T A C A C G T C T G A | 1e-98 | -227.137797 | 76.46% | 69.19% | motif file (matrix) |
| 2 | 0.729 | A G T C A G T C G C T A C A G T C T G A A G T C A T G C A C T G | 1e-95 | -220.097773 | 32.96% | 25.82% | motif file (matrix) |
| 3 | 0.622 | A G T C A C T G C G T A A G C T G A T C G C A T A G T C G C T A | 1e-85 | -195.957687 | 49.85% | 42.42% | motif file (matrix) |
| 4 | 0.634 | T A G C T C A G G C A T G C T A A G T C A T C G C G A T C G T A | 1e-82 | -188.961717 | 48.88% | 41.59% | motif file (matrix) |
| 5 | 0.672 | A T G C T G A C A T C G G C T A C A G T G C T A A G T C G A T C C G A T A G C T | 1e-57 | -131.263916 | 10.00% | 6.74% | motif file (matrix) |
| 6 | 0.690 | G C A T A G T C C T A G G C A T G C A T T G C A C A T G G T A C A C T G A C T G T A C G G T C A C A G T | 1e-55 | -128.614664 | 44.84% | 38.91% | motif file (matrix) |
| 7 | 0.608 | A T G C C T A G C T A G C A T G C G T A C T G A T C G A A G C T | 1e-50 | -116.607370 | 40.49% | 34.96% | motif file (matrix) |
| 8 | 0.646 | T C G A C T G A C A T G G T C A G T C A T C G A G A T C C T A G | 1e-48 | -110.976837 | 41.02% | 35.61% | motif file (matrix) |
| 9 | 0.618 | G C A T C G A T G T C A G T A C G T A C A T C G T A G C A T C G A C G T A C G T | 1e-43 | -99.330436 | 32.64% | 27.82% | motif file (matrix) |
| 10 | 0.609 | C A G T T G C A G A C T G C T A T G A C T C G A A G T C A C T G | 1e-35 | -80.860069 | 12.48% | 9.57% | motif file (matrix) |
